# The advent of plant cells in bioreactors

**DOI:** 10.3389/fpls.2023.1310405

**Published:** 2023-12-12

**Authors:** Fuensanta Verdú-Navarro, Juan A. Moreno-Cid, Julia Weiss, Marcos Egea-Cortines

**Affiliations:** ^1^ Bioprocessing R&D Department, Bionet, Parque Tecnológico Fuente Álamo, Fuente Álamo, Spain; ^2^ Genética Molecular, Instituto de Biotecnología Vegetal, Universidad Politécnica de Cartagena, Cartagena, Spain

**Keywords:** SIP bioreactor, single-use bioreactor, plant cell culture, secondary metabolites, recombinant proteins

## Abstract

Ever since agriculture started, plants have been bred to obtain better yields, better fruits, or sustainable products under uncertain biotic and abiotic conditions. However, a new way to obtain products from plant cells emerged with the development of recombinant DNA technologies. This led to the possibility of producing exogenous molecules in plants. Furthermore, plant chemodiversity has been the main source of pharmacological molecules, opening a field of plant biotechnology directed to produce high quality plant metabolites. The need for different products by the pharma, cosmetics agriculture and food industry has pushed again to develop new procedures. These include cell production in bioreactors. While plant tissue and cell culture are an established technology, beginning over a hundred years ago, plant cell cultures have shown little impact in biotechnology projects, compared to bacterial, yeasts or animal cells. In this review we address the different types of bioreactors that are currently used for plant cell production and their usage for quality biomolecule production. We make an overview of *Nicotiana tabacum*, *Nicotiana benthamiana*, *Oryza sativa*, *Daucus carota*, *Vitis vinifera* and *Physcomitrium patens* as well-established models for plant cell culture, and some species used to obtain important metabolites, with an insight into the type of bioreactor and production protocols.

## 1 Introduction

Recently, new ways to produce vaccines and medicinal compounds are appearing and taking center stage. Time and again, global emergencies foster advances in science and technology. Obtaining a product with pharma quality means that its manufacturing complies with the Good Management Practice (GMP) as defined by the Food and Drug Administration. Early work in the nineties showed the feasibility of producing functional antibodies in plant cells (Ma et al., 1995). The subsequent technology development brought the concept of molecular farming using whole plants to produce molecules for the health industry (Fischer and Emans, 2000; Fischer and Buyel, 2020).

The manufacturing of molecules for vaccinating or health purposes, requires a production environment that is much easier to achieve in a bioreactor, or cell lysate than in a greenhouse. The rationale behind using whole plants to produce vaccines and proteins for health security is based on several assumptions (Ma et al., 1995; Twyman et al., 2003). First generating plants producing an exogenous protein is relatively straight forward, second plants can be amplified with ease, thus upscaling is a matter of having more plants via seeding larger surfaces. Despite the initial hype, it has taken nearly two decades to have the first plant produced protein with an FDA approval in the market, and it is produced in bioreactors (Tekoah et al., 2015a).

While plant molecular farming has solved many important steps, for protein production, high quality secondary metabolite production is also a promising field. However, how to grow plant cells for a GMP process or simply for the highest quality product is not straight forward. Whilst bioreactors can be considered highly engineered farms for cell production, they are not well known by plant biotechnologists. In this review we addressed plant cell cultures in bioreactors. We give an overview of the different types of bioreactors currently used with their advantages and disadvantages. We also describe the most representative plant species used in the industry for cell culture. We give a short overview of some representative species used to obtain high quality metabolites and the culture procedures currently used.

## 2 Plant cell cultures: current state and future perspectives

### 2.1 Recent advances in plant cell cultures in bioreactors

A bioreactor is a controlled environment designed to support the growth, cultivation, and manipulation of living organisms, such as cells, or microorganisms, under specific and optimal conditions. It is commonly used in various fields including biotechnology, pharmaceuticals, and research to produce biological products, conduct experiments, or study biological processes. Bioreactors provide controlled parameters such as temperature, pH, nutrient supply, and oxygen levels to optimize the growth and production of desired biological substances.

Recent advances in plant cell cultures in bioreactors encompass genetic engineering for metabolite production, microcarrier optimization for enhanced growth, or 3D culture systems mimicking natural environments (Hu, 2020; Hesami et al., 2021). Real-time monitoring and automated control of culture conditions is performed using different sensors such as pH or conductivity (Ruffoni et al., 2010; Wang et al., 2016). Precise control over environmental factors such as light intensity in the case of photobioreactors, or temperature, and dissolved oxygen has been implemented for a long time (Ruffoni et al., 2010; Zhao et al., 2015). Furthermore, synthetic biology is used for novel pathways, nutrient formulation enhancement, and integration of omics technologies with bioreactor data for a holistic understanding of cellular processes. (Ochoa-Villarreal et al., 2016; Reski et al., 2018). These technologies are collectively driving more efficient and productive plant cell culture processes with applications in pharmaceuticals, nutraceuticals, and other valuable compounds.

Scaling up plant cell cultures from laboratory volumes to industrial bioreactors has been one of the major focuses. New strategies for maintaining uniform growth include nutrient distribution, and dissolved oxygen. Plant cells are especially sensitive to mechanical shear stress due to agitation. Thus improvements are being developed, allowing for more efficient and cost-effective production of plant-derived compounds (Georgiev et al., 2013; Motolinía-Alcántara et al., 2021). One drawback associated with plant cell cultures is their extended cultivation process duration when compared to microbial cultures, primarily owing to the comparatively sluggish growth rate of plant cells. To address this challenge, the development of process strategies, such as semi-continuous and/or continuous perfusion processes, has been undertaken to bolster productivity (Zhong, 2002; Corbin et al., 2020). Unless the cell itself is the final product, cell growth is not the main outcome of the process, but a minimum growth is required to obtain any meaningful production (see below).

Researchers have been investigating strategies to induce stress responses in plant cells, which can trigger the accumulation of secondary metabolites with potential therapeutic benefits. Bioreactor conditions can be manipulated to simulate various stress factors and enhance metabolite production (Zhang et al., 2012; Narayani and Srivastava, 2017; Ashraf et al., 2018; Ho et al., 2020).

Plant cell suspension cultures, derived from undifferentiated callus cells, are established through the disaggregation of cells originating from delicate calli in liquid media. To efficiently produce recombinant proteins, an ideal plant cell suspension culture system should display rapid growth, easy genetic transformation, high protein expression capacity, minimal inherent proteolytic activity, and low levels of compounds such as phenolics or interfering phytochemicals such as oxalic acid. Additionally, the system should thrive under optimal conditions for adaptation and scalable growth within a bioreactor. While tobacco (*Nicotiana tabacum*) and rice (*Oryza sativa*) are extensively studied for generating recombinant protein through suspension cultures, other species like tomato and ginseng have also been employed in this context (Huang and McDonald, 2009).

**Figure 1:**
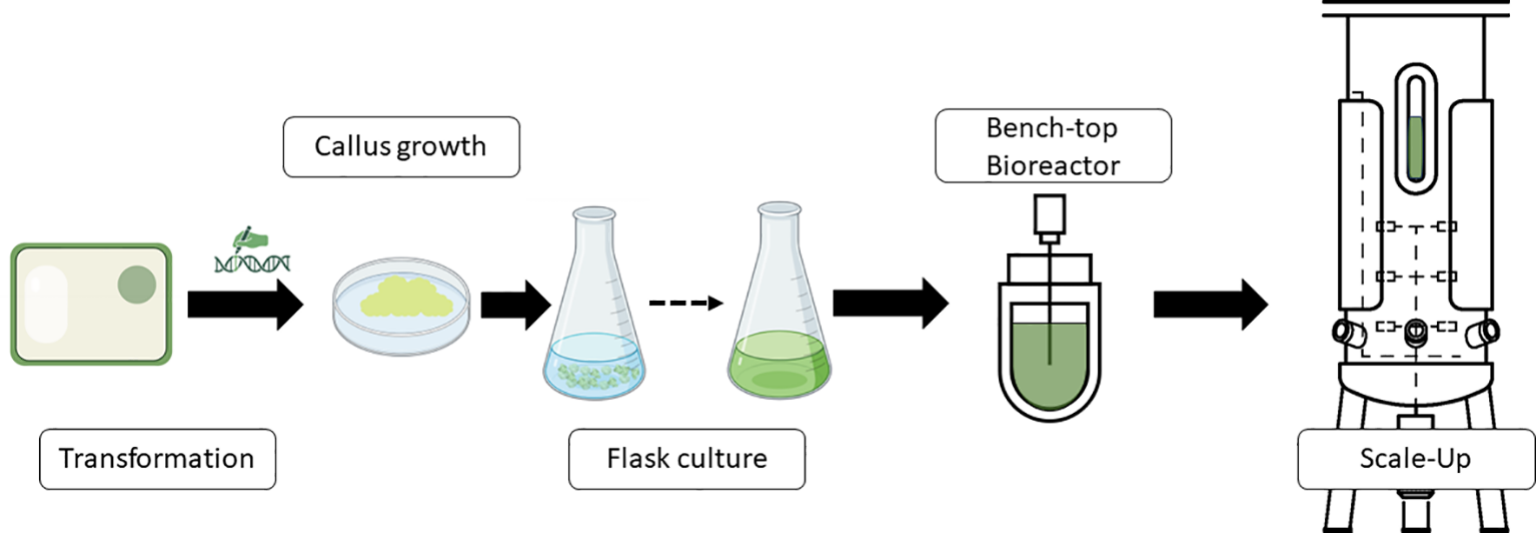
Schematic representation of a standard workflow to produce recombinant proteins or other products of interest in bioreactors industrially. Diagram was made with Powerpoint.

One typical procedure of steps to produce molecules of interest in a bioreactor using plant cells is shown in **Figure 1**. Thus, transgenic cells are produced to obtain active molecules for vaccine formulations, antibodies, or other proteins. Transgenic calli are analyzed for the presence of the transgene and expression of the protein or product if the system used is a classic overexpression construct. Once the best performers are screened, a second step begins whereby friable calli are converted into cell cultures. Once the small-scale flask-type screening of the genetic engineering steps has been developed, the bioreactor scale-up would be carried out to define the optimal requirements for process parameters, automation and monitoring needs, bioreactor design, and parameters for scaling up the process.

The specifications of the bioreactor used to obtain the final product may differ but the previous operations of the biological material preparation before the bioreactor step will be similar in most cases.

### 2.2 Bioreactor types for plant cell cultures

**Figure 2:**
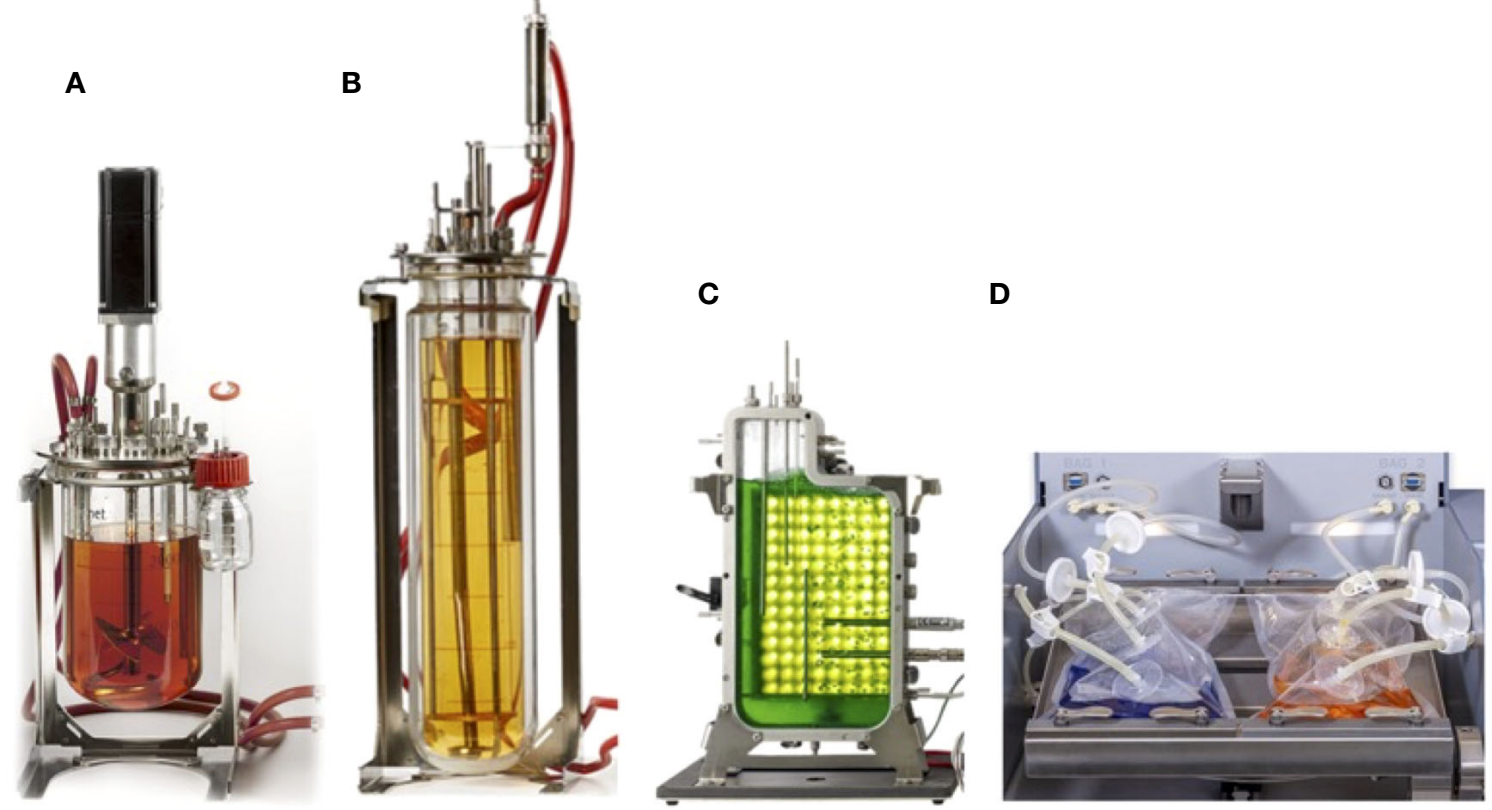
Range of typical laboratory-scale bioreactors for plant cell culture **(A)** continuous stirred bioreactor with cell culture configuration, **(B)** airlift bioreactor without mechanical agitation, **(C)** flat-panel photobioreactor design, and **(D)** single-use bioreactor with 2D rocking system.

**Figure 3:**
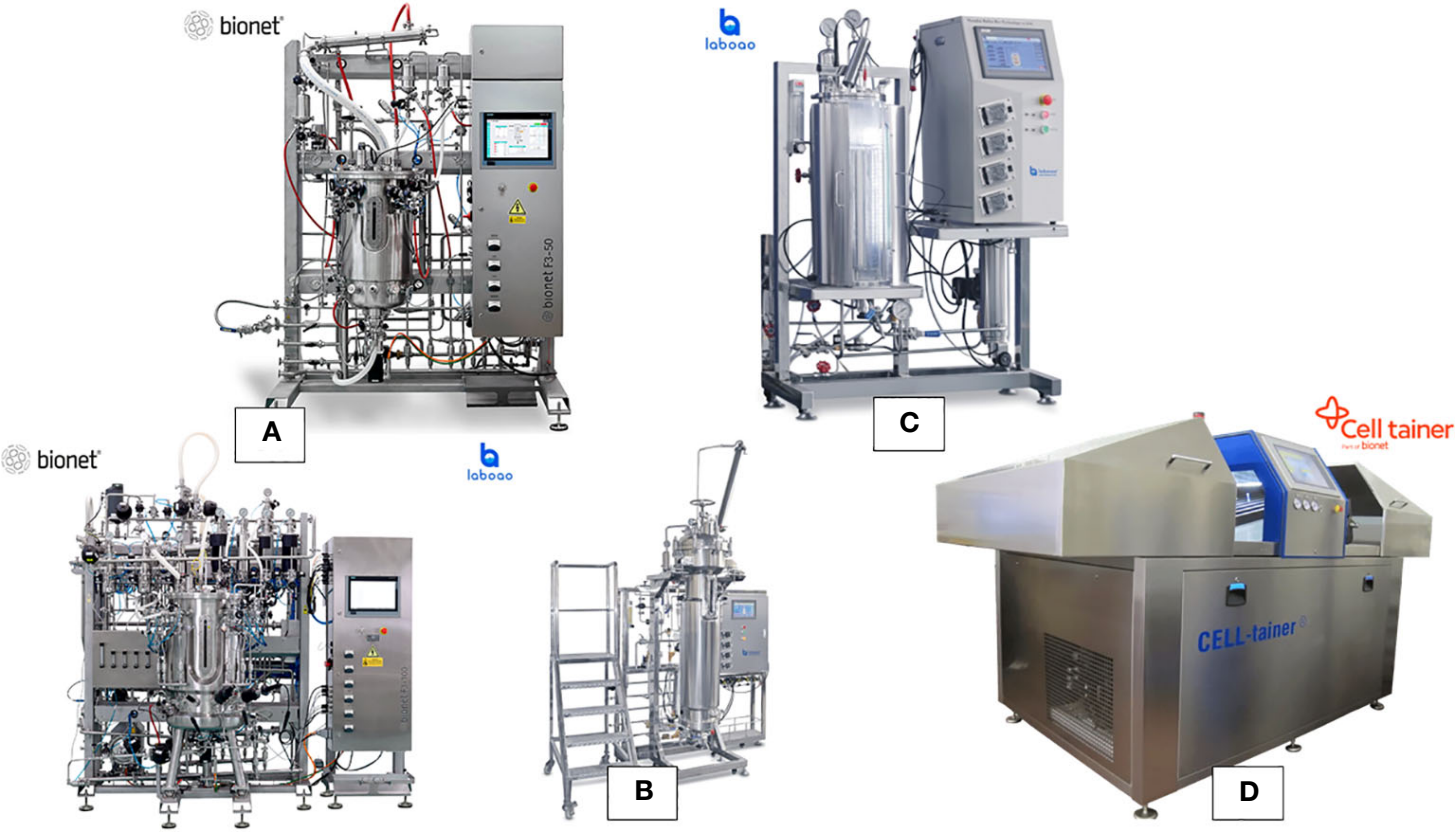
Range of the typical bioreactors at pilot-industrial scale for plant cell culture **(A)** stainless steel continuous stirred bioreactors with cell culture configuration, **(B)** stainless steel airlift bioreactor, **(C)** stainless steel bioreactor with illumination (photobioreactor), and **(D)** single-use bioreactor with 2D rocking system.

This section provides an in-depth exploration of various bioreactor types employed for plant cell cultures, showcasing their characteristics, applications, and contributions to advancing plant cell biotechnology. Bioreactors are used across a range of scales, including a laboratory for research and optimization (**Figure 2**), the pilot for process validation and scaling up, and an industrial for large-scale production of plant cells and their products (**Figure 3**).

Bioreactors can be classified based on preparation and sterilization methods. We identify three categories:

- Autoclavable bioreactors are typically constructed from glass with volumes ranging from 200 mL to 10 liters. They undergo full sterilization within an autoclave, encompassing probes and often culture medium. Larger volumes are unpractical as finding an autoclave accommodating a large flask is not easy. They become increasingly cumbersome to handle and may brake during manipulation.
- Sterilization in-place (SIP) bioreactors, constructed mainly of stainless steel with capacities ranging from 15 liters to 500 cubic meters. They are sterilized using heat and steam pressure, often along with probes and medium.
- Single-use bioreactors (SUB), inherently sterile, are made from plastic. They are usually gamma irradiated or treated with ethylene oxide. They come equipped with sterile probes, and the medium can be conventionally sterilized separately through heat or microfiltration or procured pre-sterilized commercially. The scale of working volumes ranges from 1mL-100 mL in the case of microscales, or 100mL-10L for bench-top scale, and 10-200 L for the pilot -scale up to 2000L for commercial scale.

#### 2.2.1 Stirred-tank reactors

**Figure 4:**
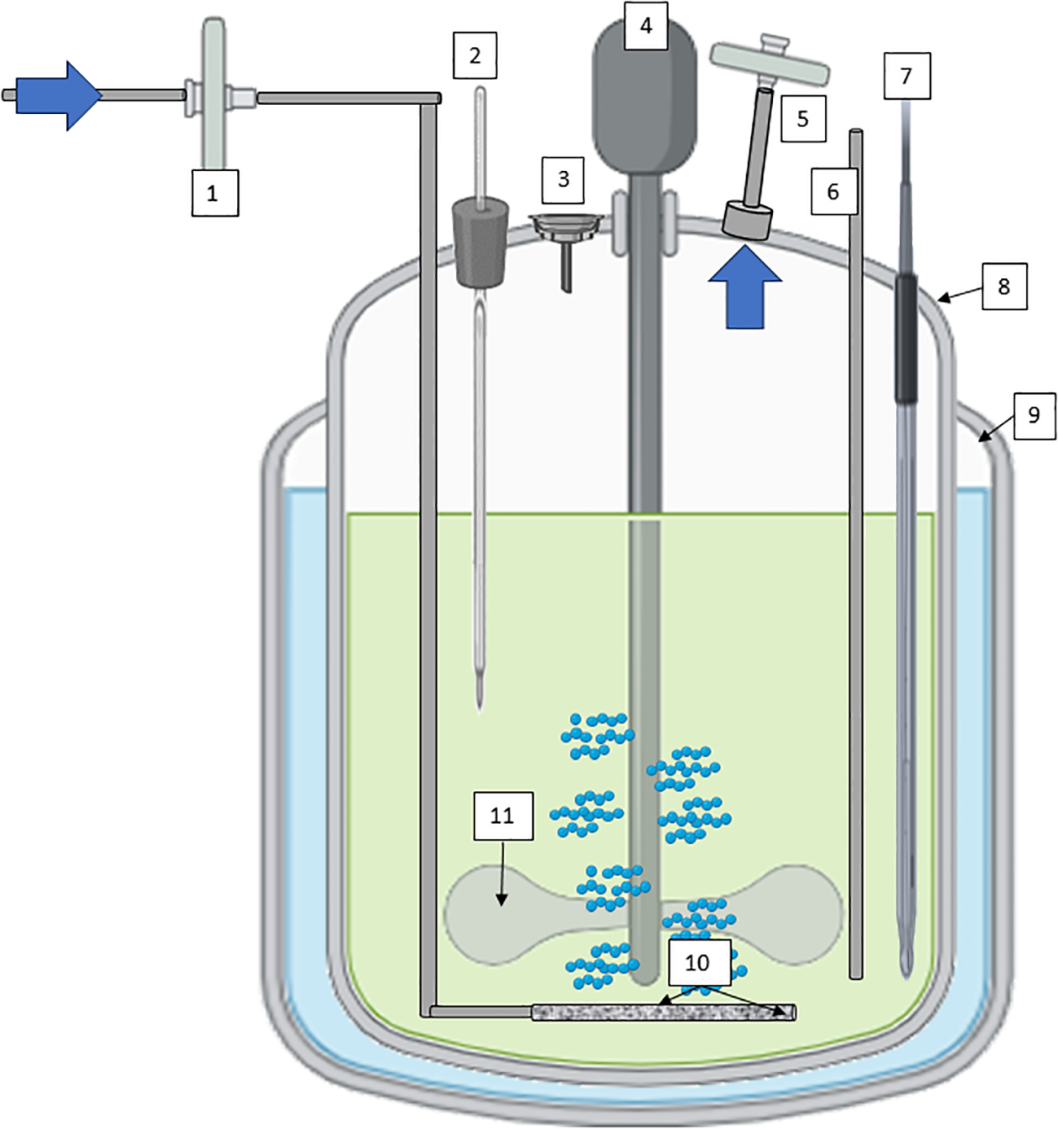
Diagram of a CSTR with mechanical agitation for plant cells cultivation; 1) sterile filtration inlet gas, 2) Temperature probe, 3) Inoculation/addition port, 4) low stirring speed motor, 5) exhaust line with filter, 6) harvest line, 7) pH/conductivity/pO2 probes, 8) vessel geometry 2:1, 9) jacket for temperature maintenance; 10) microsparger, and 11) impeller at low shear-rate effect. Diagram was made with Powerpoint.

Continuous Stirred-tank bioreactors (CSTR), often used in suspension cultures, offer efficient mixing through mechanical agitation, promoting uniform nutrient distribution and gas exchange (**Figure 4**). Their versatility spans both research and large-scale production, with features like controlled temperature, pH, and dissolved oxygen levels enhancing cell growth and metabolite production (Eibl and Eibl, 2008; Ruffoni et al., 2010). The scalability of stirred-tank bioreactors makes them the working horse in industrial settings, where reproducibility and yield optimization are paramount. Several secondary metabolites obtained by plant cell culture suspension are produced using CSTR technology. Paclitaxel (Taxol) derived from Taxus spp. (Yew Tree) cell cultures is an anticancer compound used in chemotherapy (Tabata, 2004; Frense, 2007). Continuously stirred bioreactors are used to produce this compound for pharmaceutical purposes. Artemisinin derived from *Artemisia annua* (sweet wormwood) cell cultures is used as a treatment for malaria. Continuous cultivation can increase the yield of artemisinin for pharmaceutical applications (Baldi and Dixit, 2008; McCoy and O’Connor, 2008). Other compounds of interest obtained by cell suspension of plants with CSTR are industrial enzymes such as papain, which is a proteolytic enzyme, or bioactive compounds with potential health benefits, such as terpenoids (ginsenosides), polyphenols such as ellagic acid from Grafaria cultures with antioxidant properties, or saponins (soyasaponins).

In the case of CSTRs, all three methods of preparation and sterilization are available. These are: autoclave, steam in place and single use, unlike other types of bioreactors, that have some design or technical limitations.

#### 2.2.2 Airlift bioreactors

Airlift bioreactors are a type of pneumatically agitated bioreactor that uses a combination of gas sparging and fluid circulation to create a continuous flow of nutrients, oxygen transfer, and maintenance of homogeneous temperature, without mechanical agitation. Suspension cultures of *Berberis wilsoniae* are used to produce alkaloids, and the formation of the phenolic alkaloids depends on the concentration of dissolved oxygen. In case of *B. wilsoniae*, a considerable yield increase results from the higher aeration rates by using an airlift bioreactor (Breuling et al., 1985). The production of ginseng cells applies ALB in the absence of mechanical agitation in order to avoid mechanical shear stress but at low aeration rates (Thanh et al., 2014). As in the case of ginseng cells, some of the ALBs used in root-forming plant cell processes may have special balloon-like configurations instead of the standard 8:1 or 10:1 height/diameter geometries. Other examples of such ALBs are found in the production of bioactive phytochemical compounds such as *Hypericum perforatum* L. (St. John’s Wort) (Cui et al., 2014).

#### 2.2.3 Photo-bioreactors

While plant cells can happily grow in media with sugars, salts, and micronutrients, plus the right combination of plant hormones, algae do not need sugars or hormones but light. Photobioreactors are specialized bioreactors designed to cultivate photosynthetic microorganisms, such as algae and cyanobacteria, using light as the primary energy source for growth and production (Bergmann and Trösch, 2016; Sathinathan et al., 2023). Mosses can grow efficiently in PBR with minimum media in autotrophic conditions (Hohe and Reski, 2005; Beike et al., 2010). Though, their yields can be increased by supplementing the basal medium with microelements (ME), sucrose at low concentrations, ammonium nitrate or tartrate (Hohe and Reski, 2002; Beike et al., 2015). In the case of sucrose addition, the mosses have a mixotrophic metabolism.

These PBR can be vertical to maximize light exposure and space utilization by arranging plant cells in vertically stacked layers. This design is suitable for high-density cell culture and can be used for plant cell production. Another type is tubular PBR, consisting of long transparent tubes through which the culture medium flows. This design offers efficient light exposure and can be adapted for plant cell culture to enhance biomass production. To solve high-density cultures and light limitations, the flat panel PBR is a solution. Flat panel PBR is often used for microalgae cultivation but can be adapted for plant cells. These systems consist of thin, flat containers with transparent surfaces for light penetration, allowing for efficient growth in a compact space. Finally, outdoor PBR implements these types of bioreactors in outdoor environments for plant cell cultivation, specially algae (Lee et al., 2014). These systems take advantage of natural sunlight and can be used for the large-scale production of plant cells for various applications. In the case of single use bioreactors with 2D rocking system, as they are transparent bags, lights can be installed inside the equipment, which is another PBR option.

#### 2.2.4 Single-use bioreactors

Single-use bioreactors have gained popularity due to their ease of setup and reduced risk of cross-contamination. There are several types of single-use bioreactors for plant cell cultivation. As commented previously, continuous stirred-tank bioreactors (CSTR) feature a rotating impeller that gently stirs the culture medium, ensuring even mixing and oxygen distribution. Rocking systems, do not have stirring agitation. Arguably it could be an optimal mixing system for plant cells cultures that form aggregates or can suffer from shear-stress. They offer a more flexible approach to plant cell culture in bioreactors, especially for smaller-scale applications (Georgiev et al., 2013; Oosterhuis and Junne, 2016; Jossen et al., 2019).

**Figure 5:**
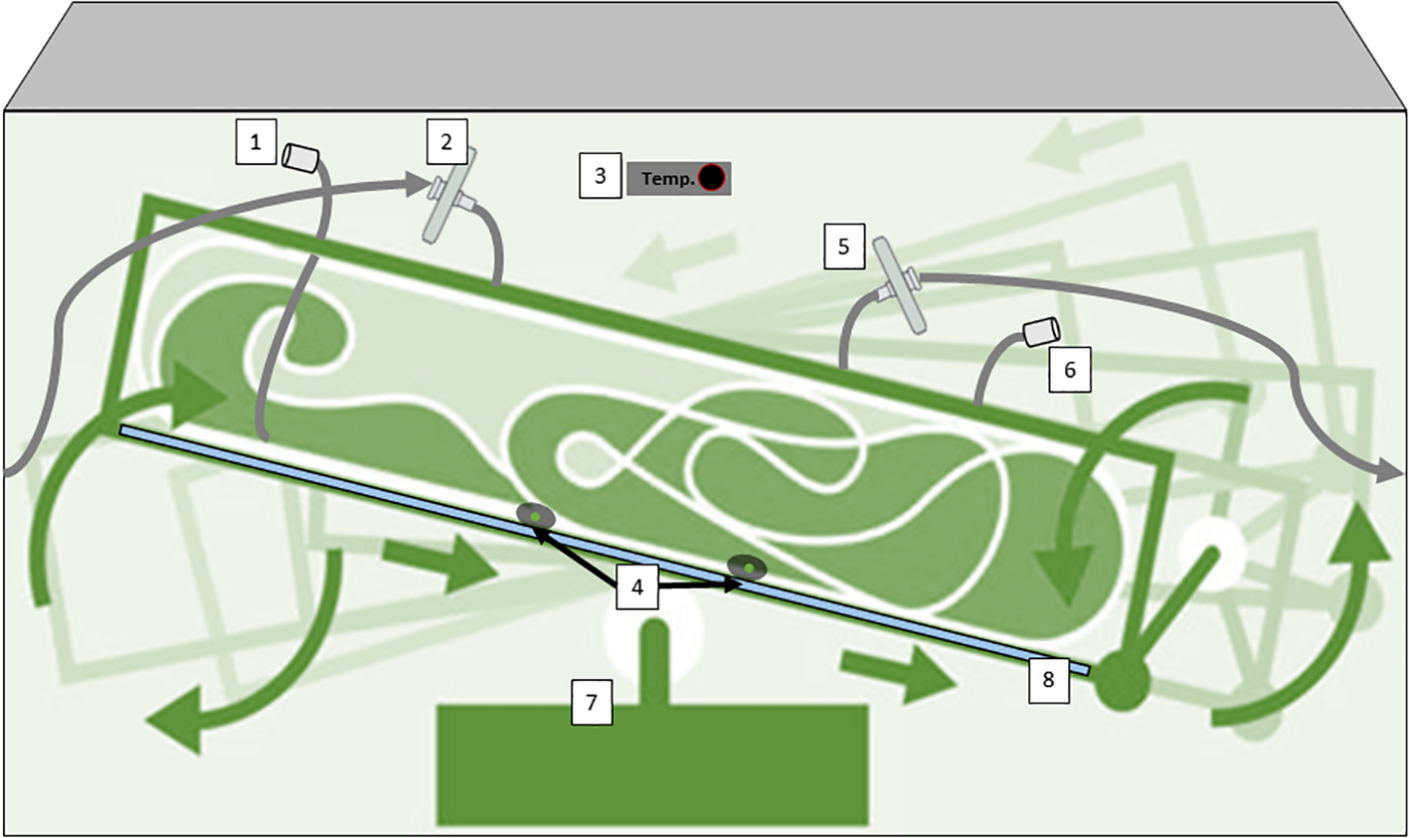
Diagram of a Single-Use bioreactor with 2D rocking system for plant cells cultivation (CELL-tainer®); 1) harvest line,2) sterile filtration inlet gas, 3) Temperature probe, 4) pH/conductivity/pO2 probes, 5) exhaust line with filter, 6) Inoculation/addition ports, (7) rotor, (8) cooling plate. This device has a patented mixing technology. Diagram was made with Powerpoint.

Wave-induced bioreactors use wave-like motions to create a similar effect, and their disposable nature eliminates cleaning (Bartczak et al., 2022). Additionally, 2D rocking bioreactors sway the culture back and forth, promoting nutrient and gas exchange (Eibl and Eibl, 2008; Eibl et al., 2011). These diverse options offer efficient, contamination-resistant environments for growing plant cells in various scales of research and production. In the case of 2D rocking, single-use bioreactors for plant cells are bags with two rocking motions that simulate the movement of the waves of the sea. This 2D movement enhances mass transfer, oxygenation, and nutrient distribution, improving plant cell growth and productivity (Oosterhuis and Junne, 2016). Equipped with advanced controls and aseptic features, these bioreactors provide an efficient and scalable platform for cultivating plant cells and producing valuable plant-derived products. One example is the Cell-tainer® bioreactor which is based on a 2-D rocking system that achieves high productivity with a variety of cell types including microalgae, bacteria, and fungi (**Figure 5**). (Oosterhuis and van den Berg, 2008; Zijlstra and Oosterhuis, 2010; Hillig et al., 2014). Commercially we find single-use industrial systems that use bags with an ALB-type aeration system, such as the production of the therapeutic protein Taliglucerase alfa by the company Protalix with the ProCellEx™ bioreactor, which has been used to grow cell cultures derived from several different plant species, including *Nicotiana tabacum* (tobacco) and *Daucus carota* (carrot) (Tekoah et al., 2015b).

### 2.3 Advantages and disadvantages of different types of bioreactors

Various types of bioreactors, including Continuous Stirred-Tank Reactors (CSTR), single-use bioreactors (SUB), airlift bioreactors (ALB), and photobioreactors (PBR), offer distinct advantages and disadvantages in the production of plant cells. **Table 1** illustrates the unique characteristics of each bioreactor type. The selection of the most suitable bioreactor depends on several factors, such as the specific requirements of the plant cell culture, the nature of the product, scale considerations, and available resources (Merchuk, 1990; Shukla and Gottschalk, 2013).

**Table 1:** Advantages and disadvantages of the main types of bioreactors for the cultivation of plant cells.

Bioreactors Type	Advantages	Disadvantages
CTSR (autoclavable/sterilizable glass vessels or stainless-steel material)	**Steady-State Operation**: Continuous operation, maintaining a steady-state environment for consistent cell growth and product formation. **Controlled Conditions**: Precise control over temperature, pH, and nutrient levels can be achieved. **Scalability**: Easy scale up for larger production volumes. **High mixing Efficiency**: Uniform nutrient distribution and prevents gradients within the reactor.	**Limited Light Availability**: Not suitable for photosynthetic cultures. **High Energy Consumption** **Shear Stress** **Contamination Risk**: Continuous operation increases the risk of contamination, which is critical in plant cell cultures.
ALB (autoclavable/sterilizable glass vessels or stainless-steel material)	**Low Shear Stress** **Improved Oxygen Transfer** **Scalability**: Scalable and can be adapted to different production volumes. **Reduced Contamination Risk**: Closed systems reduce the risk of contamination compared to open systems.	**Non-Uniform Mixing**: Mixing can be less uniform compared to other systems. **Higher Initial Investment** **Complex Design**: **Difficult Process Control**: Achieving precise control over culture conditions can be challenging due to varying flow patterns.
PBR (autoclavable/sterilizable glass or transparent material)	**Direct Light Utilization**: Photobioreactors are ideal for photosynthetic cultures, utilizing light energy efficiently for growth and product synthesis. **High Productivity**: Optimized light exposure can lead to high biomass and product yields **Carbon Capture**: Algae in photobioreactors can absorb carbon dioxide, contributing to carbon capture and potential environmental benefits. **Minimal Contamination**: Closed systems reduce the risk of contamination from external sources.	**Complex Design**: Photobioreactor design can be complex, requiring careful consideration of light distribution and gas exchange. **Light Limitation**: Light penetration can limit cell growth in deeper layers of the culture. **Maintenance of Light Conditions**: Ensuring consistent light exposure across the culture can be challenging. **Initial Investment:** Setting up photobioreactor systems with proper lighting can involve higher initial costs.
SUB (stirred, wave, and 2-D rocking systems)	**Reduced Cleaning and Sterilization**: **Flexibility:** Different batches or cell lines can be cultured successively without extensive cleaning, allowing for rapid process changeovers. **Lower Cross-Contamination Risk**: The disposability of the bioreactor bag reduces the risk of cross-contamination between batches. **Lower Initial Investment**: Setup costs can be lower due to reduced infrastructure and equipment requirements.	**Limited Scalability**: Scaling up single-use bioreactors involves using multiple units, which could be less efficient and more costly. **Environmental Concerns**: The disposal of single-use plastic components raises environmental concerns. **Limited Control over Reactor Design**: Users may have limited customization options for specific process needs. **Pressure Limitations**: Single-use systems might have pressure limitations, restricting their use for certain applications.

Ideally, a particular culture should undergo testing in multiple independent systems to determine optimal conditions and establish the production process. Economic factors and downstream processing requirements, as well as final attributes like Good Manufacturing Practices (GMP) compliance and cost per milligram, among others, significantly influence the choice of bioreactor. It is important to note that the investment in hardware necessary for setting up Sterile In-Place (SIP) bioreactors can be substantially higher, often by several orders of magnitude, compared to SUB or benchtop bioreactors. Therefore, the decision regarding the technology to use is usually driven by a combination of overall product needs and market pricing.

At present, the primary limiting factor in plant cell cultures is productivity. Consequently, larger volumes are typically favored, unless the produced molecule is required in very small quantities.

### 2.4 Challenges and future directions

#### 2.4.1 Mass transfer and nutrient limitations

Mass transfer limitations, particularly oxygen and nutrient availability, pose significant challenges in plant cell cultivation. As cultures grow, the demand for oxygen and nutrients may exceed their supply, leading to reduced cell proliferation and product formation. Overcoming mass transfer limitations often requires optimizing bioreactor design, agitation, and aeration strategies. Additionally, nutrient imbalance and accumulation of byproducts can impact cell health and product quality. Addressing these challenges involves refining media formulations and feeding strategies to maintain optimal nutrient levels and promote efficient metabolism (Eibl and Eibl, 2008).

#### 2.4.2 Modeling and scale-up challenges

Translating laboratory-scale plant cell cultures to larger bioreactors presents scale-up challenges. Processes that work well in small-scale cultures may not necessarily perform similarly at larger volumes due to differences in mass transfer, hydrodynamics, and cell behavior. Developing reliable mathematical models, standardized commercial designs and scaling strategies is essential for successful bioprocess commercialization. Models that account for complex interactions between cells and their environment can guide scale-up efforts and reduce the time and costs associated with process development (James and Lee, 2001; Ochoa-Villarreal et al., 2016; Motolinía-Alcántara et al., 2021).

#### 2.4.3 Regulatory considerations and biosafety

In the realm of pharmaceuticals, nutraceuticals, and the production of valuable compounds using plant cell cultures, the importance of regulatory considerations and biosafety concerns cannot be overstated. To ensure product excellence, uniformity, reproducibility in manufacturing, and overall safety, strict adherence to regulatory directives is imperative. This includes the stringent Good Manufacturing Practice (GMP) standards upheld by the pharmaceutical sector.

It is worth noting that projects originating from academic environments often encounter regulatory obstacles that could be preemptively avoided by implementing specific procedures, such as GMP, right from the outset of the process.

Furthermore, the potential for unintended genetic modifications or cross-contamination underscores the need for meticulous oversight to prevent inadvertent consequences. Regulatory bodies play a pivotal role in shaping the progress and commercialization of bioreactor-based plant cell production systems. This encompasses the development of robust process control and monitoring mechanisms, including associated software applications (Murthy et al., 2015).

According to Agrios, 2005 book “Plant Pathology,” Plant Biotechnology is defined as the application of tissue culture and genetic engineering techniques to create genetically modified plants with new or improved characteristics. However, alternative definitions exist that do not involve genetic modification. The United States Department of Agriculture characterizes it as a set of techniques employed to tailor plants for specific needs or opportunities. Hall, 1984, describes it as a study of methods to customize plant resources for industrial processes and novel materials used in agriculture, forestry, and horticulture.

It is evident that plant biotechnology is not meant to replace traditional agriculture but rather complement it to address the growing global demands. This extends beyond enhancing crop yields or resistance to environmental and biological stresses. Plant biotechnology aims to develop plants capable of producing pharmaceuticals, vaccines, and other high-value industrial products—a concept known as molecular farming in plants.

Despite the potential benefits of this application, it faces opposition. Some segments of society express concern about genetically modified organisms, fearing potential environmental degradation and reduced biodiversity (Rifkin, 1998; Food Biotechnology in Ethical Perspective, 2007). Another point of contention revolves around the perceived injustice in the distribution of intellectual property rights (IPR). Skeptics argue that corporations might monopolize natural resources, creating potential inequities (Heffernan, 1999; Timmermann, 2020).

The current feeling is that large corporations and monopolies raise when harsh regulatory environments impose lengthy processes from product to the market as only strong companies with financial resources can survive the time required before starting to introduce a new product. Our view is that many products for the food and cosmetic industries will be developed directly from plant tissues without requiring transgenic approaches.

## 3 Cell types used in bioreactors

The use of plant cell cultures for different purposes started first by having a reliable protocol to obtain them. Thus, some platforms have been used in many different experimental setups due to well established transformation protocols and cell lines. Most of the early studies in bioreactors and cell cultures have been done with *Nicotiana tabacum* BY2 cells. A second group of cells include plant cell cultures that produce a single molecule of high interest and are not engineered (see below).

### 3.1 Nicotiana tabacum

While tobacco cells have a proven track record in academic research, they show important liabilities when it comes to producing high quality pharmaceutical biomolecules. This has led to a fruitful research and development to improve it as a protein production platform. Modified glycosylation has a positive impact on the cytotoxic activity of recombinant antibodies. This has led to the engineering of the glycosylation pathway in Chinese Hamster ovary cells (Umaña et al., 1999). A similar strategy has been developed in plant cells. Efforts have included the inactivation of a fucosyl transferase and a glycosil transferase to obtain human-like glycosylation (Mercx et al., 2017; Smargiasso et al., 2019; Herman et al., 2021). The presence of endogenous proteases also decreases both quality and quantity of protein production and RNAi strategies have been used to downregulate endogenous proteases with some success (Mandal et al., 2014). These proteases as expected are found in other plant genomes such as *N.benthamiana* or *Arabidopsis thaliana* (Magy et al., 2014). Thus, knowledge obtained in *N. tabacum* may be useful for other plant cell platforms. Whilst the reduction of endogenous proteases by genetic means is a formal possibility, a second strategy is the use of modified culture conditions. Indeed, increased pH and the generic serine and cysteine protease inhibitor AESBF showed significant improvements on the stability of alpha antitrypsin (rATT) (Huang et al., 2009). The addition of exogenous generic protease inhibitors such as BSA, gelatin, PEG and others have been studied with mixed results as they have short life times, and they may interfere with downstream processing and have a negative effect on cell growth (Benchabane et al., 2008; Huang and McDonald, 2012). A strategy with a built in protease inhibitor has been reported with improved protein production in potato leaves (Rivard et al., 2006).

Despite being a less than perfect platform, tobacco cells have an impressive record of production of different proteins. Early proof of concepts demonstrated the ability to produce antibodies, Interleukin 2 and 4 in tobacco cells (*N. tabacum*) (Magnuson et al., 1996l Magnuson et al., 1998). Importantly, both IL2 and IL4 are biologically active. Another example of antibody production in tobacco cells (BY-2 cells) is the antibody M12. It binds to vitronectin, a glycoprotein found in the serum and extracellular matrix (Raven et al., 2015). It is used to fight against cancer. The bioreactor used was a 200L disposable device. The human granulocyte-macrophage colony-stimulating factor (GM-CSF) was also produced using the BY-2 cells (James et al., 2000). Next to these examples, the number of proteins produced in tobacco cell cultures goes up to several hundred. And current efforts include a holistic approach to improve growth media, bioreactors, cell properties and promoters.

### 3.2 Nicotiana benthamiana

The wild tobacco plant *Nicotiana benthamiana* has become an important model system in plant biology. The genome of *N. benthamiana* shows an allotetraploid structure. Several genome versions have been published (Bombarely et al., 2012; Schiavinato et al., 2019; Kurotani et al., 2023). Work performed in *N.benthamiana* started as a model system for plant pathogen interactions (Goodin et al., 2008). However, early work showed its potential to study gene function by Virus Induced Gene Silencing (Liu et al., 2004). In fact *N.benthamiana* has a defective gene silencing pathway, allowing high expression of viral vectors as it is highly susceptible to viral infection (Bally et al., 2018). This is an advantage to produce very high quantities of exogenous proteins using viral vectors.

The complete plant of *N. benthamiana* has been extensively used to produce many proteins and metabolites. The process has been mostly based on agroinfiltration using T-DNA constructs or via viral vector infiltration of leaf tissues. A variety of proteins have been produced in *N.benthamiana* leaves (Alkanaimsh et al., 2016; Jiang et al., 2020; Mateos-Fernández et al., 2021). However, like in the case of *N.tabacum*, the endogenous proteases are a big hurdle to obtain high productions (Grosse-Holz et al., 2018; Jutras et al., 2020).

In contrast to BY2 cells derived from *N.tabacum*, use of *N.benthamiana* in liquid cultures is less extended. By using a knockdown strategy of β-1,2-xylosyltransferase and α-1,3-fucosyltransferase, in combination with the expression of an anthrax decoy fusion protein, this product was successfully generated in 1L flasks (Sukenik et al., 2018). By applying the mannosidase I inhibitor kifunensine, the anti-CD20 rituximab has been obtained in transient expression with no fucosylation, displaying a 14 fold increase of antibody-dependent cell-mediated cytotoxicity (Kommineni et al., 2019). This indicates that *N. benthamiana* is a good platform that may take over *N. tabacum* as choice for protein production.

### 3.3 Rice cells

Rice is probably the most important crop in the world. Working with rice has several advantages including a very well defined genome (Goff et al., 2002; Huang et al., 2012) with an excellent annotation (Ouyang et al., 2007; Sakai et al., 2013; Ren et al., 2019). Many rice varieties have been sequenced allowing the identification of structural variants (Fuentes et al., 2019).

Rice cell culture protocols were established more than 30 years ago (Mikami and Kinoshita, 1988; Torres et al., 1999), and ever since, they have been used for research and biotechnological purposes. Due to the importance of rice as a crop, comprehensive studies have been performed to identify genes involved in callus formation, a prerequisite for robust transformation protocols (Zhang et al., 2019). These include traits such as induction rate, induced callus weight, induced callus color, induced callus size, friability, callus proliferation ability, induction speed, and callus first appearance. Whilst plant transformation is generally genotype dependent, rice cultivars *of O.sativa japonica* and *indica* appear to have decent callus formation and regeneration capacity (Abe and Futsuhara, 1986; Binte Mostafiz and Wagiran, 2018).

Rice cell cultures have been used to produce many proteins. These include human serum albumin (Pang et al., 2020), lysozyme (Huang et al., 2002) butyrylcholinesterase (Corbin et al., 2016; Macharoen et al., 2020) or human α-1-Antitrypsin (McDonald et al., 2005).

### 3.4 Carrot cells

Carrot cells have a long tradition in *in vitro* culture. They have pioneered studies to express foreign proteins such as chitinase, or the GLUTAMIC ACID DECARBOXYLASE autoantigen 65 (Gilbert et al., 1996; Porceddu et al., 1999). However, these studies used whole plants. Carrot cells have also played a role in uncovering the importance of cell density in cultures as a major factor impacting survival (McCabe et al., 1997). The successful production of the taligluciferase alfa with comparable activity to Cerezyme® produced in CHO cells paved the way for industrial production (Shaaltiel et al., 2007). The recombinant plant-derived GCD (prGCD) is targeted to the storage vacuoles, using a plant-specific C-terminal sorting signal from *Arabidopsis* endochitinase and a storage signal from tobacco endochitinase. Further development by Protalix (Tekoah et al., 2015a) allowed it to become the first plant produced biotherapeutic approved by regulatory authorities.

### 3.5 Physcomitrium

The moss *Physcomitrium patens*, previously known as *Physcomitrella* (Medina et al., 2019), has emerged as a valuable model system in plant science, primarily due to its significant advantages for genome manipulation. The complete genome of *Physcomitrium*, encompassing nuclear, plastid, and mitochondrial genomes, has already been published (Lang et al., 2018; Perroud et al., 2018).

Utilizing this moss species as a model system offers several notable benefits for plant functional genomic studies, particularly its ability to incorporate exogenous DNA into the genome through homologous recombination (Schaefer, 2002). *Physcomitrium* exhibits remarkable gene targeting efficiency and shares structural and functional similarities with higher plants, making it an excellent choice for studying plant biology. Being haploid, means that identifying phenotypes is straightforward (Egener et al., 2002).

Moreover, the cultivation of *Physcomitrium* in bioreactors is highly advantageous due to its inherent genetic stability and remarkable tolerance to fermentation parameters such as shear stress. To maximize protein production yields, it is recommended to culture the moss using filamentous protonema tissue. Maintaining the culture at pH levels within the range of 4.5-6 is crucial during this specific stage of the life cycle (Decker and Reski, 2012).

However, not all that glitters is gold for *Physcomitrium* cultures, as they face certain hurdles. Being autotrophic, they do not require a medium with carbon sources, but they are dependent on light. This can pose challenges in terms of bioreactor configuration and the space required to accommodate the light source. Additionally, these cultures demand extended cultivation periods of at least 20-30 days to achieve high yields, especially when operating in continuous or semi-continuous mode. Maintaining the filamentous protonema tissue in long-term cultures is crucial, but this can be effectively achieved through pH control during bioreactor cultivation (Hohe et al., 2002).

One company that specializes in producing pharmaceuticals and therapies based on *Physcomitrium* cultures is Eleva GmbH, formerly known as Greenovation Biotech GmbH. They have successfully developed treatments for various diseases, such as C3 Glomerulopathy, Fabry disease, and Pompe disease (Häffner et al., 2017; Hennermann et al., 2019; Hintze et al., 2020; Hintze et al., 2021). Their production process is carried out in single-use wave bioreactors of up to 200L capacity (Niederkrüger et al., 2019).

### 3.6 Plant cells for tailored molecule production

The plant chemodiversity is being exploited to produce many chemicals with a wide range of industrial purposes. While traditional industrial processes relayed on extraction from cultivated or wild species, this practice is not sustainable (Payne and Shuler, 1988). Especially when endangered species with high value secondary metabolites are not well-established culture plants.

#### 3.6.1 Grapevine (*Vitis* spp.)

The grapevine stands as a significant crop with diverse applications. Beyond its role as a delectable fruit, it holds paramount importance in the creation of wine and vinegar. Yet, within its clusters lies a concealed treasure: an abundant store of phenolic compounds. These natural metabolites function as potent antioxidants, bestowing remarkable health advantages upon humans. This phenomenon, famously illustrated by the “French Paradox” (Sun et al., 2002; Lippi et al., 2010; Hu et al., 2015; Galinski et al., 2016), underscores the grapevine’s significance.

Numerous studies have been undertaken to amplify the production of these invaluable compounds. The augmentation of sucrose content, for instance, has yielded escalated levels of anthocyanins (Pirie and Mullins, 1976; Hirasuna et al., 1991). Additionally, pre-treatment with benzothiadiazole has exhibited the capacity to enhance both anthocyanin and resveratrol synthesis, concurrently conferring acquired resilience to *Botrytis cinerea* (Iriti et al., 2004).

Grapevine has emerged as a promising model for eliciting culture as a means of enhancing the production and accumulation of valuable secondary metabolites, notably resveratrol. Conventionally, this elicitation process involves the utilization of chemical elicitors, such as methyl jasmonate and jasmonic acid. These compounds are naturally synthesized by plants and play a key role in plant responses to both biotic and abiotic stresses. When introduced to grapevine cell cultures, these molecules simulate stress conditions, thereby activating the production of secondary metabolites (S et al., 2016).

Lijavetzky et al. achieved remarkable extracellular yields of trans-resveratrol by employing a combination of methyl jasmonate and cyclodextrins. Notably, cyclodextrins function as elicitors and additionally serve as carriers for trans-resveratrol, effectively encapsulating the compound. This encapsulation phenomenon alleviates the constraints on trans-resveratrol production, leading to heightened accumulation of this secondary metabolite (Almagro et al., 2011).

Subsequently, this process has been successfully upscaled in bioreactors to facilitate the production of resveratrol and its derivatives, including viniferins. This scaling has been implemented in both batch and fed-batch modes, even reaching capacities of up to 20 liters (Donnez et al., 2011; Nivelle et al., 2017; Chastang et al., 2018; Lambert et al., 2019).

#### 3.6.2 Withania somnifera

The so-called indian ginseng or *Withania somnifera* is a plant producing withanone, withanolide A and withaferin A, among other compounds (Sivanandhan et al., 2013). These compounds have a large number of target molecules in humans having great potential for different therapies. Production of the main metabolites *in vitro* culture has theoretical advantages over field grown plants (Kaur et al., 2022). However, complete pharmacological production has not been fully achieved.

#### 3.6.3 Catharantheus roseus

*Catharantheus roseus*, commonly known as Madagascar periwinkle, is a plant species known for its production of terpenoid indole alkaloid metabolites, some of which hold significant pharmaceutical potential. Notably, certain metabolites exhibit promising antitumor and anti-hypertensive properties (Contin et al., 1999). Among these metabolites, catharantine stands out for its remarkable antitumor activity. Researchers have discovered that low doses of UV-B radiation can induce and enhance catharantine production in cell cultures of this species (Ramani and Chelliah, 2007). Additionally, various biotic elicitors have demonstrated effectiveness in boosting catharantine yields, even in bioreactor cultures (Zhao et al., 2000a; Zhao et al., 2000b; Zhao et al., 2001a; Zhao et al., 2001b; Almagro et al., 2015b).

Other strategies for alkaloid production are centered around two-stage batch cultures (Drapeau et al., 1986; Payne et al., 1988). This approach involves an initial growth phase to attain high biomass levels, followed by a metabolite production phase. Typically, the medium in the bioreactor needs to be changed between these stages. However, there’s also the possibility of achieving metabolite production using a single production medium, resulting in a one-stage culture (Morris, 1986; Zhao et al., 2001a).

Beyond elicitation and two-stage cultures, there are additional avenues to enhance metabolite yields, with metabolic engineering being one of the most promising. This approach entails the manipulation of biosynthetic pathways and transcription factors, among other techniques (Zhao and Verpoorte, 2007).

However, the outlook for industrial-scale production of indole alkaloid metabolites from *Catharantheus roseus* is somewhat pessimistic at present due to persistently low yields. Combining the methods mentioned above may be necessary to achieve the desired results.

#### 3.6.4 *Taxus* spp.

The Taxus genus is renowned for its production of valuable medicinal metabolites, with the most famous one being paclitaxel, commercially known as Taxol®. Paclitaxel plays a vital role as an anti-tumor agent in chemotherapy. Since the traditional methods aren’t as effective, producing paclitaxel using cell cultures are the most promising method (McElroy and Jennewein, 2018; Zhu and Chen, 2019). Nowadays, Phyton Biotech is a leading provider of paclitaxel to the global market (Wilson and Roberts, 2012).

The primary method for enhancing paclitaxel and other taxane production involves elicitation through both abiotic and biotic elicitors (Vongpaseuth and Roberts, 2007; Onrubia et al., 2010; Ramirez-Estrada et al., 2015; Ramirez-Estrada et al., 2016; Vidal-Limon et al., 2018). It’s worth noting that attempts to produce taxanes in bioreactors date back to earlier work (Vanĕk et al., 1999; Son et al., 2000; Navia-Osorio et al., 2002).

#### 3.6.5 *Lithospermum* spp.

Shikonin is a versatile molecule known for its antibacterial, anti-inflammatory, and natural dye properties (The Pharmacopoeia of Japan, 1982). It is produced by species in the Boraginaceae family, with Lithospermum being one of them. Studies have successfully scaled up the production process in bioreactors up to 750L and at high cell densities (Tabata and Fujita, 1985; Tanaka, 1987). The two-stage culture system mentioned earlier is employed in this process.

#### 3.6.6 Apple tree

Remarkably, high-value metabolites can be obtained even from common plant species like apple trees. For instance, apple cell cultures have been used to produce a compound that proves highly useful in treating a deadly disease—trypanosomiasis (Andre et al., 2022). This molecule is a 3-O-p-coumaroyl ester of tormentic acid. The metabolites were produced in stirred tank bioreactors with up to 4L working volume. The process can be scaled-up to industrial volumes.

#### 3.6.7 Plant cells for biodiversity

Plant cell cultures can be used for production of rare biomolecules produced by plants. Plant chemodiversity is enormous and many plants found their way into traditional medicine. Many of these plants may be under thread as they are not cultivated, but rather gathered from the wild (Nalawade et al., 2003). A large number of studies have been published where different plants have been produced *in vitro* to ensure propagation of rare genotypes and ensure their survival (Zhou and Wu, 2006). Some examples include *Psoralea coryfolia* to produce daidzein and genistein (Shinde et al., 2009), galanthamine production by *Crinum malabaricum* (Priyadharshini et al., 2020; Chahal et al., 2022) or *Eryngium alpinum* to produce phenolic compounds (Kikowska et al., 2020). However, in most cases, a protocol to obtain cell cultures for bioreactor production has not been developed, suggesting that it may be a good opportunity for future development.

## 4 Comparing plant cells with other platforms for the biotechnology industry

The development of high quality biomolecules by biotechnological processes involving genetic engineering can be traced back to the mid-seventies with the production of somatostatin and insulin in *E.coli* as a major breakthrough (Itakura et al., 1977; Goeddel et al., 1979). This breakthrough showed that active peptides can be produced in heterologous cells, starting the era of genetic engineering and biotechnology. As the genetics of *E.coli* are well understood, improved cells have been engineered to obtain high quality proteins. Most *E.coli* strains used for protein production have been obtained by improvement of the *E.coli* BL21(DE3) strain (Miroux and Walker, 1996). This strain, has loss of function mutations in proteases (LON and ompT) (Mizusawa and Gottesman, 1983; Grodberg and Dunn, 1988). Further genetic improvements include tightly regulated promoters, such as the T7 RNA polymerase promoter (Doherty et al., 1993), codon usage optimization and up to rafinesse such as improved disulfide bond formation via mutations in thioredoxin reductase (Proba et al., 1995). Currently titers of 10g/L of high quality protein can be obtained with *E.coli* strains, see (Rosano et al., 2019) for a recent review.

*Komagataella phaffii*, formerly known as *Pichia pastoris*, represents a yeast strain of significant relevance within industrial protein synthesis. This particular species finds extensive application as a host for heterologous protein production and has garnered widespread attention in scientific literature (Cregg et al., 2000; Macauley-Patrick et al., 2005; Cregg et al., 2009; Juturu and Wu, 2018; Barone et al., 2023). Its prominent attributes encompass the ability to conduct high cell density fermentations using well developed and scalable technologies, rapid and easily automated genetic modification (Vogl et al., 2013). It has post-translational modifications characteristic of eukaryotes (Qin et al., 2011; Meehl and Stadheim, 2014), remarkable efficacy in secretion and biomass production (Weinacker et al., 2013). It shows genetic constructs of enduring stability (Goncalves, 2013), and an increasingly diverse range of tools readily available to the public (Lin-Cereghino et al., 2013; Weinacker et al., 2013; Ahmad et al., 2014; Ergün et al., 2021). Several notable therapeutics have been obtained utilizing this system, including enzymes like alpha-galactosidase used in Fabry disease treatment, the hepatitis B vaccine antigen HBsAg, and insulin analogs (Cregg et al., 1987; Kjeldsen et al., 1999; Chen et al., 2000; Hardy et al., 2000). Additionally, Pichia-produced biologics such as monoclonal antibodies targeting diseases like cancer or autoimmune disorders, growth factors like granulocyte colony-stimulating factor (G-CSF) (Pykhtina et al., 2020; Gautam et al., 2021), and antifungal proteins have shown promise in therapeutic applications (Virágh et al., 2014; Popa et al., 2019). The platform’s ability to generate complex proteins with proper folding, post-translational modifications, and scalability has made it a valuable tool in producing various biotherapeutics for clinical use (Daly and Hearn, 2005; Puxbaum et al., 2015; Juturu and Wu, 2018).

Heterologous production of proteins in Chinese Hamster Ovary (CHO) cells, started soon after *E.coli*, with obtention of dihydrofolate reductase deficient mutants (DHFR) allowing stable transformation (Urlaub and Chasin, 1980). Early successes such as the production of interferon (Scahill et al., 1983), and the adaptation of stirred bioreactor technology from *E.coli*, allowed a rapid improvement of CHO, in similar ways to those of *E.coli*. These include optimized bioprocess and transgene expression, and a big effort to engineer protein export machinery in the cells (Kuo et al., 2018).

In contrast to the aforementioned production platforms, plant biotechnology has not developed a single cell of choice that has been optimized for heterologous protein production. Rather, different approaches and partially redundant efforts in different species appear to compete. Furthermore, a major effort has gone into producing exogenous proteins in whole plants in the so-called molecular farming approach (Fischer and Buyel, 2020). As a result, plant cell culture biotechnology is less well developed. Interestingly the first plant-produced product sold in the market is made in carrot cell cultures (see above). Plant cell cultures, have their own niche in the production of rare chemicals by a very large diversity of plants. However, sheer productivity is a liability of cell cultures as in most cases they have not been optimized to have economically relevant yields. This is an important field for future development.

## 5 Improving production via multistep processes

Induction is a fundamental concept in microbial and cell culture, facilitating the controlled expression of target molecules, such as recombinant proteins or metabolites, at specific times and in specific quantities. It involves the activation of a promoter that governs the transcription of the target gene (Weake and Workman, 2010).

In microbial expressions, classic examples include bacteria like *Escherichia coli* and yeasts such as *Saccharomyces cerevisiae* or *Pichia pastoris* (Gossen et al., 1994; Chen, 2012; Baghban et al., 2019). In *E. coli*, the widely used promoter is the lac promoter, which regulates genes involved in lactose metabolism within the lac operon (Hannig and Makrides, 1998; Kilikian et al., 2000). In *P. pastoris*, the alcohol oxidase 1 (AOX1) promoter is a common choice. To express a target gene, it’s inserted downstream of the AOX1 promoter in a Pichia expression vector, with methanol serving as the inducer (Macauley-Patrick et al., 2005). More recently, alternative promoters to AOX, such as GAP (glyceraldehyde-3-phosphate dehydrogenase), have emerged due to safety concerns in the methanol-based induction process (García-Ortega et al., 2019).

In the case of yeast *S. cerevisiae*, controlling gene or protein expression often involves genetic manipulation. For instance, a target gene can be inserted under the control of the GAL (galactose-inducible) promoter. The addition of galactose to the growth medium activates the GAL promoter, leading to the expression of the gene of interest (Flick and Johnston, 1990).

In mammalian cell culture, induction methods include adding specific chemical inducers or controlling culture conditions. The tetracycline-inducible system relies on adding tetracycline or its analogues to the culture medium to activate the expression of a target gene. Alternatively, gene expression in mammalian cells can be induced by controlling factors like temperature or other environmental conditions (Gray, 1997; Romanova and Noll, 2018).

In plant cell cultures, the biosynthesis or enhancement of specific compounds can be influenced by various biotic or abiotic elicitors. Biotic elicitors involve interactions with microorganisms, enzymes, or the addition of specific hormones to the medium, while abiotic elicitors include the addition of chemicals or alterations in the culture’s physical environment. These elicitors can trigger the expression of genes related to plant growth, secondary metabolite production, or other desired traits (Namdeo, 2007).

### 5.1 Elicitors and secondary metabolite production in plant cell cultures

The use of elicitors as molecules that activate secondary metabolite pathways, has been a long-standing practice in plant biotechnology since 1992 (Gundlach et al., 1992). Subsequent research has demonstrated their efficacy in various plant types and tissues, leading to enhanced secondary metabolite production in cell cultures for biotechnological applications (Sohn et al., 2022). Particularly, the combined application of jasmonates and cyclodextrins has shown synergistic effects in activating these pathways (Lijavetzky et al., 2008), resulting in elevated levels of anthocyanins and stilbenes (Lijavetzky et al., 2008; Belchí-Navarro et al., 2012; Almagro et al., 2015a).

Amongst the secondary metabolites of interest, anthocyanins and resveratrol/stilbenes have been studied in many systems, especially cells from grape berries. The use of grape cell cultures has made it a model system to obtain and improve resveratrol and anthocyanin biosynthesis for biotechnological production (Hirasuna et al., 1991; Belhadj et al., 2008; D’Onofrio et al., 2009; Martínez-Márquez et al., 2016; Jeong et al., 2020). Moreover, other elicitors, such as coronatine, a fungal elicitor that binds to the JA receptor COI-1, have also been employed to improve secondary metabolite production (Almagro et al., 2015a; Lee et al., 2018).

However other biotic elicitors include acetyl salicylic acid, salicylic acid, yeast extract, chitosan or pectin see (Alcalde et al., 2022) for a recent review. Amongst the abiotic elicitors UV radiation has been extensively used to increase anthocyanin production in planta and catharantin in *Catharantus roseus* cell cultures (Ramani and Chelliah, 2007; Liu et al., 2017; Castillejo et al., 2021; Tripathi et al., 2021).

Methyl Jasmonate (MeJa), initially identified in jasmine flowers by Demole et al., 1962, serves as a pivotal phytohormone alongside its free-acid counterpart, jasmonic acid, participating in various plant processes. The primary function of MeJa and jasmonic acid lies in activating plant defense mechanisms against both biotic and abiotic stresses (Wasternack and Parthier, 1997). Notably, the jasmonates receptor is shared with the phytotoxin coronatine, the COI1 (Feys et al., 1994; Xie et al., 1998). Both coronatine and jasmonates initiate similar molecular pathways, such as anthocyanin accumulation, illustrating the versatility of these compounds beyond biotic stress responses. Abiotic stresses, also trigger secondary metabolites production pathways like anthocyanin biosynthesis (Christie et al., 1994; Rudolf and Resurreccion, 2005; D’Alessandro et al., 2022).

Salicylic acid, identified as another elicitor by Angelova et al., 2006, plays a significant role in the plant defense pathway (Chen et al., 1995; Ding and Ding, 2020). No other phytohormones are known to act as elicitors, since they do not participate in the same way in plant defense as they do.

Expanding on this notion, molecules beyond traditional phytohormones can act as elicitors to trigger plant defense pathways. In recent years, there has been a notable shift towards using nanoparticles (NPs) instead of biotic elicitors, owing to the customizable physicochemical properties of NPs. Metallic, metal oxide, combinations of metallic NPs, magnetic, silica, and biopolymer NPs represent diverse categories that have demonstrated promising elicitation results and increased production yields in *in vitro* cultures (Fazal et al., 2016; Jamshidi and Ghanati, 2017; Javed et al., 2018; Nourozi et al., 2019; Khan et al., 2021; Arya et al., 2022).

Although using JA as a method is a simple and effective way to increase secondary metabolite production, it has some important caveats. First, JA is a stress signal and as such, it is not a good procedure for fed-batch cultures as stressing the cells causes a decrease in overall growth capacity. This can be overcome in some cases by increasing sugar input (Kümmritz et al., 2016). In general, elicitors activate a stress response that causes an inhibition of cell division (Logemann et al., 1995). Thus, when elicitors are used, we create a two-step production protocol of growth followed by activation of secondary metabolite production at a cost of increased biomass.

## 6 Conclusions

While early work using whole plants opened the pathway for molecular farming in plants, current advances indicate that there is a bright future for plant cell cultures to produce high added value proteins, VLPs and metabolites for the health, veterinary, cosmetic and food industry. The nature of the bioreactor environment is not new to the pharmaceutical industry that relies heavily on animal cells, *E. coli* and *Pichia pastoris* for production. It is a matter of time that new products will be enter the market using plant cells as a chassis of choice. The current coalescence of synthetic biology, improved bioreactor design and an increased number of plant cell platforms is opening new production opportunities.

The future of plant cell cultivation lies in the continuous advancement of bioreactor technology, coupled to a synthetic biology approach for product optimization. Emerging trends include the integration of artificial intelligence and machine learning for real-time process optimization, the development, and implementation of single-use bioreactors for enhanced scalability and reduced contamination risk, the incorporation of sensors for automated monitoring and control, or the development of different operation modes and strategies to increase productivity.

These challenges and future directions collectively shape the trajectory of plant cell cultivation in bioreactors. Overcoming these obstacles and capitalizing on emerging trends will play a pivotal role in the sustainable and efficient production of valuable compounds from plant cells.

Concerning cell types, there are currently two opposite trends. Plants chemodiversity may be better exploited by obtaining a chemical component out of its original plant produced in a bioreactor. In contrast, the continuous improvement of proven chassis cells such as carrot, *N. benthamiana*, rice or *Physcomitrium* may be advantageous. Having a proven type of cell that is accepted by the pharma industry is a step forward to produce many future products in a GMP format using plant cells.

Metabolic engineering of distinct pathways in plants has been achieved targeting different metabolites. One distinct advantage of CRISPR technologies is the possibility of targeting multiple paralogs in a genome. Increased isoflavones have been obtained in soybean by simultaneously targeting *GmF3H1*, *GmF3H2* and *GmFNSII-1* (Zhang et al., 2020). The obtention of tomatoes enriched in gamma aminobutyric acid (Li et al., 2018). By modification of different genes, recent advances have shown the enrichment between 4 to 1500 fold in the concentration of naringenin, eriodictyol, kaempferol, and quercetin in leaves of *N. benthamiana* (Selma et al., 2022). These works show that metabolic engineering has a bright future, and it is a matter of time that these processes will find their way into plant cell cultures. Whilst plant cells can produce many different compounds, the same technology can be applied in other systems with success. Indeed, anthocyanins have been produced in E.coli (Cress et al., 2017), and complex metabolic pathways to synthesize opium alkaloids and cannabinoids have been successfully transferred to the yeast *S. cerevisiae* albeit with low productivities(Galanie et al., 2015; Luo et al., 2019). This suggests a future competition for a platform that can deliver the highest titers of a given compound, and this may not always be in a plant cell.

## Author contributions

FV: Conceptualization, Formal Analysis, Funding acquisition, Writing – original draft, Writing – review & editing. JM: Conceptualization, Supervision, Writing – original draft, Writing – review & editing, Funding acquisition. JW: Conceptualization, Funding acquisition, Project administration, Supervision, Writing – original draft, Writing – review & editing. ME: Conceptualization, Funding acquisition, Project administration, Supervision, Writing – original draft, Writing – review & editing.
